# Imaging features of recently identified low-grade vascular neoplasia of the liver: hepatic small vessel neoplasm and anastomosing hemangioma

**DOI:** 10.1007/s00330-025-11915-4

**Published:** 2025-09-04

**Authors:** Maïté Lewin, Rauda Aldhaheri, Aurélie Beaufrère, Christophe Desterke, Anita Paisant, Ivan Bricault, Paul Borde, Gabriel Simon, Mickaël Lesurtel, Daniel Cherqui, Clara Prud’Homme, Valérie Vilgrain, Astrid Laurent-Bellue

**Affiliations:** 1 Department of Radiology, Paul Brousse University Hospital, AP-HP-University Paris Saclay, Villejuif, France; 2 Department of Pathology, Beaujon Hospital, FHU MOSAIC, AP-HP University Paris-Cité, Clichy, France; 3 INSERM U1310, University Paris-Saclay, Medicine Faculty, Paul Brousse University Hospital, Villejuif, France; 4 Department of Radiology, Angers University Hospital, Laboratoire HIFIH UPRES EA3859, SFR ICAT 4208, Université d’Angers, Angers, France; 5 Department of Radiology, Grenoble University Hospital, Grenoble, France; 6 Department of Radiology, Lille Catholic Hospitals, Lille Catholic University, Lille, France; 7 Department of Radiology, CHRU Besançon, University of Bourgogne Franche-Comté, Besançon, France; 8 Department of HPB Surgery and Liver Transplantation, Beaujon Hospital, AP-HP University Paris-Cité, Clichy, France; 9 Hepatobiliary Center, Paul Brousse University Hospital, AP-HP-University Paris Saclay, Villejuif, France; 10 Department of Radiology, Beaujon Hospital, FHU MOSAIC, CRI INSERM U 1149, AP-HP University Paris-Cité, Clichy, France; 11 Department of Pathology, Bicêtre Hospital, AP-HP University Paris Saclay, Le Kremlin-Bicêtre, France

**Keywords:** Liver neoplasms, Vascular neoplasms, Magnetic resonance imaging, Diagnostic imaging

## Abstract

**Objectives:**

The aim of this study was to describe the imaging features on dynamic CT and MRI of a series of pathologically confirmed low-grade vascular neoplasia of the liver (LGVNL).

**Materials and methods:**

In this retrospective multicenter study, patients diagnosed with pathologically proven LGVNL between January 2014 and August 2024 and with cross-sectional imaging (CT or MRI) were included. Based on prior studies, we divided the patients into two groups: a group with typical LGVNL features and a group with atypical tumors. Univariable analysis and the logistic regression model were used to evaluate the outcome of typical and atypical LGVNL features.

**Results:**

Twenty-eight patients were included (20 men, mean age 53.7 ± 13.4 [SD] years old). The median size of tumors at diagnosis was 22 mm [IQR, 10–80]. A typical LGVNL pattern including thick continuous peripheral arterial phase “flower petal shape” enhancement was found on MRI in 67% (18/27) with lobulated lesions (*p* = 0.001), a marked hypersignal on T2-weighted images (*p* = 0.003) and a high apparent diffusion coefficient (ADC) (mean tumor ADC, 2.1 × 10^−3^ ± 0.3 [SD] mm^2^/s) (*p* = 0.006) with portal and delayed phase filling following contrast administration. An atypical LGVNL pattern was found in 33% (9/27), including homogeneous arterial phase enhancement that persisted during the portal and delayed phases. After a median follow-up of 16 months, tumor growth was observed in 42% (8/19) and was faster in patients with the atypical LGVNL pattern (*p* = 0.013).

**Conclusion:**

The typical imaging pattern of LGVNL that includes arterial phase “flower petal shape” enhancement with a marked hypersignal on T2-weighted images and a high ADC was seen in most cases.

**Key Points:**

***Question*** *Low-grade vascular neoplasia of the liver, including hepatic small vessel neoplasms and anastomosing hemangioma, is a new entity of vascular tumors with few radiological descriptions.*

***Findings*** *Diagnostic imaging criteria include the “flower petal shape” enhancement pattern on arterial phase, a marked hypersignal on T2-weighted images and high ADC values.*

***Clinical relevance*** *This multicenter study provides clinical, pathological and imaging data on low-grade vascular neoplasia of the liver and highlights specific imaging features for its diagnosis. Knowledge of these imaging features helps eliminate differential diagnoses.*

**Graphical Abstract:**

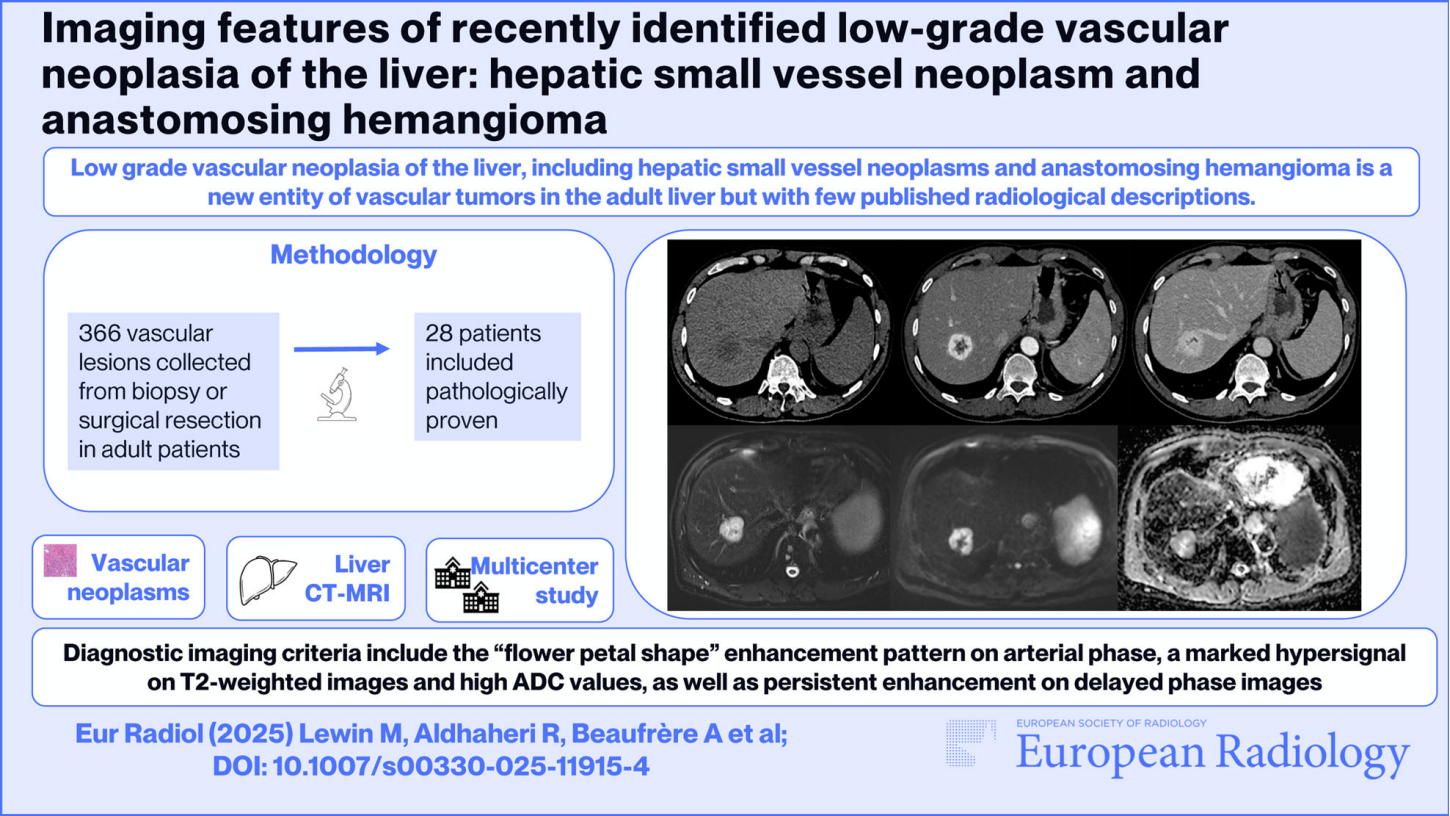

## Introduction

Vascular tumors of the liver are mesenchymal lesions that develop from endothelial cells [1]. According to the International Society for the Study of Vascular Anomalies (ISSVA) classification, hepatic cavernous hemangiomas, which are the most common liver lesions in adults with a prevalence of 3–20%, are considered venous malformations [2, 3]. Therefore, the list of the primary vascular tumors in the adult liver includes intermediately aggressive hepatic epithelioid hemangioendothelioma (HEHE) and hepatic angiosarcoma, the most aggressive liver tumor in humans [3, 4]. A new rare entity called low-grade vascular neoplasia of the liver (LGVNL), which includes hepatic small vessel neoplasms (HSVN) and anastomosing hemangiomas (AH), was recently added to the list [1, 5, 6]. Indeed, AH has been identified in various organs, including in the gastrointestinal and urogenital tracts, and publications actually consider hepatic AH to be HSVN [7–12].

LGVNL is characterized on histopathology by the presence of small, closely packed vascular channels with minimal cytological atypia and very low proliferative activity. Endothelial cells are positive for standard vascular markers. HSVN and AH, which share morphological and molecular similarities, are mainly distinguished by their periphery, which is infiltrative in HSVN and well-limited in AH. Mutations for *GNAQ* and *GNA14*, which are found in various vascular malformations and tumors, have also been frequently described in these lesions [1, 5, 13].

The prognosis of LGVNL has not been clearly defined, and there are no reports of recurrence or metastatic disease (except for one case of multifocal HSVN with synchronous secondary lesions of the spleen, which was not confirmed on histology [14]). For the moment, follow-up data are limited. Because of the infiltrative growth pattern of HSVN on histology and the unknown outcome of LGVNL, resection or at least long-term follow-up is usually recommended [1, 15, 16].

The imaging features of LGVNL have not been clearly characterized because these lesions are rare and have only been recently identified [10–17]. Furthermore, various terms have been used for the same entity, such as HSVN, AH or hepatic small vessel hemangioma [17–22]. Based on the case reports in the literature, the typical imaging features of LGVNL include a lesion with thick peripheral lobulated arterial phase enhancement or a “flower petal shape” [23]. However, there are no LGVNL imaging series. Thus, these lesions are often misdiagnosed as hepatic cavernous hemangiomas, liver metastases or even hepatocellular lesions, resulting in inappropriate management.

The aim of this study was to describe the imaging features of a series of pathologically confirmed LGVN on dynamic CT and MRI.

## Materials and methods

### Study design

**Fig. 1:**
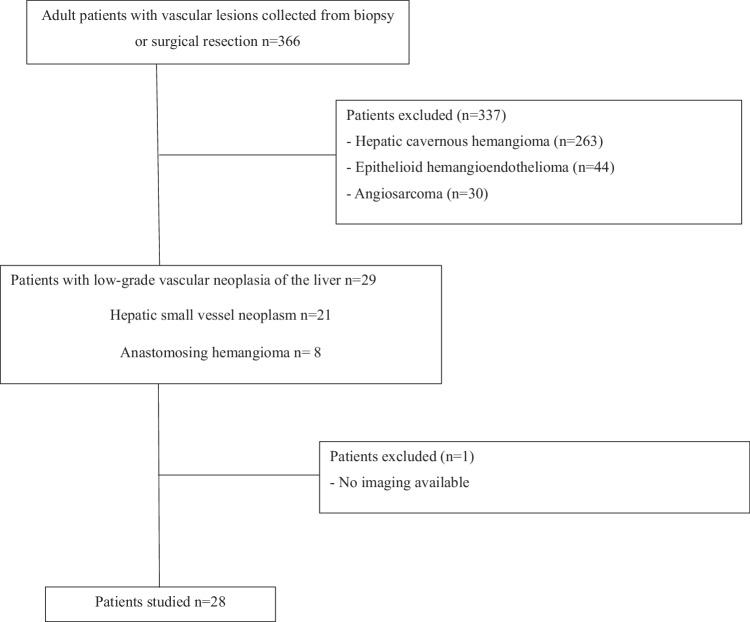
Flowchart

This retrospective multicenter study was approved by the institutional research ethics board (ClinicalTrials.gov number NCT06067035), and informed consent was obtained from all patients. The cases were identified by searching for hepatic vascular lesions from biopsies or surgical resections in the Department of Pathology from two tertiary academic centers specialized in hepatobiliary disease from January 2014 to August 2024. Patients were included with (1) histologically confirmed LGVNL, including HSVN or AH and (2) cross-sectional imaging (CT or MRI) available before biopsy or preoperatively on a PACS station. Exclusion criteria were other histologically proven vascular lesions of the liver (Fig. 1).

### Clinical data

The clinical history of patients with LGVNL was assessed according to the following data from patients’ electronic medical records: age, sex, clinical presentation at diagnosis of LGVNL (incidental findings or abdominal pain), preexisting disease such as metabolic liver disease (yes/no), cirrhosis (yes/no), history of cancer (yes/no), initial diagnosis on imaging, disease management (resection (yes/no) and follow-up).

### Histopathological review

**Fig. 2:**
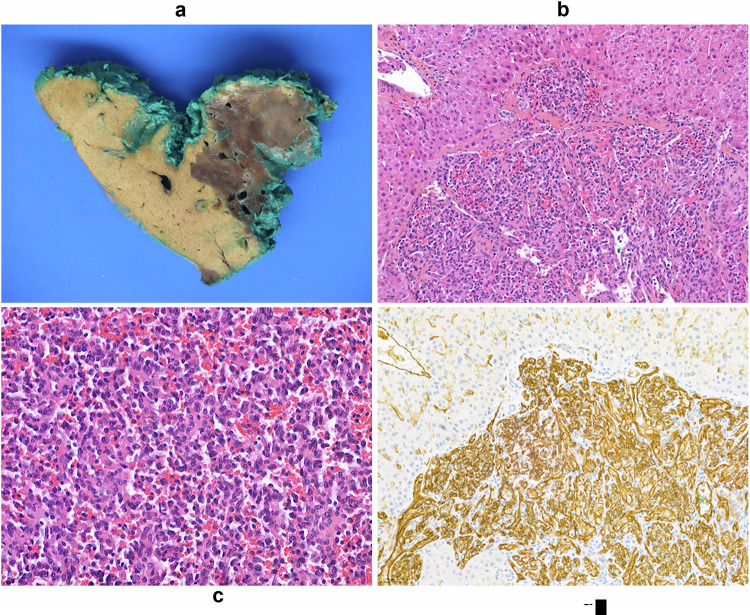
Pathology finding in a 47-year-old male with LGVNL tagged hepatic small vessel neoplasm. **a** The gross aspect of the tumor is a poorly limited gray and spongy lesion in a non-cirrhotic liver. **b** The tumor is a dense vasoformative lesion with infiltrative borders (HE original magnification × 200). **c** The tumor consists of a dense proliferation of small vessels, lined by slightly plump endothelial tumor cells without significant mitotic activity (HE original magnification × 400). **d** Immunohistochemistry results showed diffuse CD31 staining, confirming the endothelial nature of tumor cells and highlighting the infiltrative border

Slides from surgical specimens and liver biopsies were available for all patients and reviewed by two experienced liver pathologists (A.L.B., A.B.). Diagnostic criteria were based on (1) histological analysis showing that all lesions were composed of different-sized, but mainly small anastomosing vessels, no cytonuclear atypia or significant mitotic activity and (2) positive immunostaining of the following endothelial markers: ERG, CD31 and CD34 (Fig. 2). LGVNL were classified as HSVN or AH. Unlike angiosarcoma, the Ki67 index (marker of cell proliferation) was low, ranging from 2 to 5%.

### Imaging techniques

Because this was a multicenter study, CT examinations were performed with different scanners (16-to 64-row CT). A total of 23/28 patients underwent CT, and 22/23 unenhanced and multiphase contrast-enhanced CT examinations, including hepatic arterial phase (using bolus tracking software), portal phase (70–90 s), and delayed phase (180 s) images. The CT protocol of one patient did not include a delayed phase.

All patients except one underwent MRI examinations with different MR systems (1.5-T or 3-T MRI). The MRI protocol usually included T2-weighted sequences, diffusion-weighted imaging (DWI), apparent diffusion coefficient (ADC) maps, dual gradient-echo sequences (in and opposed phase), a breath-hold 3D T1-weighted fat-suppressed gradient echo pulse sequence and multiphase contrast-enhanced MRI including late-arterial, portal, and delayed phases. The patient who didn’t have a delayed phase CT underwent multiphase MRI. In addition, hepatobiliary phase (HBP) images were obtained in 15/27 patients.

Furthermore, 19 patients underwent abdominal ultrasound, and 12 underwent contrast-enhanced ultrasound (CEUS). [^18^F]FDG Positron Emission Tomography/Computed Tomography (PET/CT), ^18^F-Choline PET/CT, ^68^Ga-DOTA-TOC PET/CT, and ^18^F-FDOPA PET/CT were available in 6, 5, 4, and 2 patients, respectively.

### Image analysis

Images were analyzed by three radiologists (R.A. “in training” and two senior radiologists, M.L. and V.V., with 25 and 30 years of experience in abdominal and liver imaging, respectively). In cases of disagreement, a consensus reassessment was performed. The following features were evaluated on CT and MRI for each LGVNL: (1) tumor size (defined as the largest cross-sectional diameter); (2) number of lesions categorized as single lesion, oligo-lesions (≤ 5) or multifocal lesions (> 5), in patients with multiple lesions, the largest lesion was evaluated; (3) tumor margins classified as well defined (lobulated/ rounded) or infiltrative; (4) signal intensity (SI) on T2-weighted images categorized as moderate or marked hyperintensity (defined as ≥ that of the spleen) with or without the presence of flow voids (i.e., serpiginous vessels into the tumor); (5) SI on T1-weighted images categorized as hypointense (yes/no) compared to the adjacent liver parenchyma; (6) SI on DWI (b value > 400 s/mm^2^ up to 900 s/mm^2^) compared to the liver categorized as hyperintense or isointense; (7) tumor ADC value and the ADC ratio between the lesion and the adjacent parenchyma (ADC value of tumor/ADC value of adjacent liver parenchyma); (8) enhancement pattern during arterial phase hyperenhancement (yes/no), including the presence of a “flower petal shape,” continuous thick peripheral lobulated enhancement (yes/no), or homogeneous enhancement (yes/no); (9) portal and delayed phase enhancement categorized as present in relation to the surrounding liver or washout appearance; (10) SI in the HBP compared to the adjacent liver parenchyma categorized as hypointense to liver (yes/no); (11) the presence of a capsule, calcifications, necrosis, hemorrhage, tumor in vein or biliary dilatation (yes/no); (12) fat deposition on chemical shift imaging in the mass; and (13) tumor growth during imaging follow-up according to Response Evaluation Criteria in Solid Tumors, version 1.1. Time points for follow-up imaging were at the discretion of the clinical physician at irregular intervals. Tumor echogenicity (hyper-echoic, iso-echoic or hypo-echoic according to the adjacent liver parenchyma), enhancement on CEUS and tumor metabolism on medicine nuclear imaging were also reported if available.

Based on the imaging findings in this series and in the literature, we divided the patients into two groups: a group of patients with a lesion showing continuous thick peripheral lobulated arterial phase enhancement or a “flower petal shape” called typical LGVNL imaging pattern and a group of patients without the “flower petal shape” but with homogeneous arterial phase enhancement called atypical LGVNL imaging pattern [23].

### Statistical analysis

Statistical analyses were performed (CD) with R software environment version 4.3.3. Categorical variables and qualitative imaging features were described as frequencies and percentages. Univariable analyses for the outcomes with typical and atypical imaging features were performed for each clinical and radiological parameter with Publish R-package version 2023.01.17. Statistical significance testing was two-sided t-tests for quantitative variables and chi-square tests for qualitative variables. Starting with epidemiological and significant radiological parameters as covariates, a logistic regression model was built with “generalized linear model” (glm) R-base function (logit-family as parameter) for the outcomes with typical or atypical imaging features. The variables used to perform logistic regression analysis were age at diagnosis, clinical male gender and a marked T2 hyper-intensity. Agreement for the determination of outcome among the three radiologists was evaluated by pairs using Cohen’s kappa score with “irr” R-package version 0.84.1. Time-event analysis of size increase was performed with survival R-package version 3.7-0, and the Kaplan Meier plot was drawn with survminer R-package version 0.4.9. *p* ≤ 0.05 was significant.

## Results

### Clinical characteristics

Patients with hepatic cavernous hemangioma (*n* = 263), HEHE (*n* = 44) and angiosarcoma (*n* = 30) were excluded from the 366 adult patients with vascular lesion specimens. A total of 29 patients with pathologically confirmed LGVNL were identified with either HSVN (*n* = 21) or anastomosing hemangioma (*n* = 8); one of these patients (1 HSVN) was excluded due to an absence of imaging studies (Fig. 1).

The final population included 28 adult patients, mean age 53.7 ± 13.4 [SD] years old (Table 1). There were more men (71%, 20/28) than women (29%, 8/28). Lesions were identified incidentally on imaging in 82% (23/28) of cases (discovery after work-up for fever or extrahepatic cancer staging). Five patients (18%) presented with abdominal pain.

**Table 1:** Clinical characteristics

Characteristic	Patients (*n* = 28)	Typical LGVNL (*n* = 18)	Atypical LGVNL (*n* = 10)	*p*-value
Age (years)	53.7 (± 13.4)	53.1 (± 14)	54.8 (± 12.8)	0.75
Sex				1.00
Male	20 (71)	13 (72)	7 (70)	
Female	8 (29)	5 (28)	3 (30)	
Clinical findings				1.00
Incidental findings	23 (82)	15 (83)	8 (80)	
Abdominal pain	5 (18)	3 (17)	2 (20)	
Clinically significant background	13 (46)	7 (39)	6 (60)	0.60
Metabolic liver disease	5 (18)	3 (17)	2 (20)	
Cirrhosis (F4 stage)	3 (11)	1 (6)	2 (20)	
History of cancer	5 (18)	3 (17)	2 (20)	
Initial diagnosis on imaging				0.49
Hepatic hemangioma	9 (32)	7 (39)	2 (20)	
Inflammatory hepatic adenoma	10 (35)	6 (33)	4 (40)	
Atypical hepatocellular carcinoma	5 (18)	2 (11)	3 (30)	
Liver metastasis	3 (11)	1 (6)	2 (20)	
Cholangiocarcinoma	1 (4)	1 (6)	0 (0)	
Management				0.49
Resection	15 (54)	11 (61)	4 (40)	
Watchful waiting	13 (46)	7 (39)	6 (60)	

Continuous variable expressed as mean ± standard deviation in parentheses. Categorical variables are expressed as numbers of patients, with percentages in parentheses

*F* fibrosis

Underlying liver disease was found in 29% (8/28) of the patients, including 3 with cirrhosis, and 18% (5/28) had a history of cancer (breast cancer, small bowel neuro-endocrine tumor, gastrinoma, prostate cancer, and gastric cancer).

All lesions were initially misdiagnosed on imaging. The initial diagnosis was hepatic cavernous hemangioma in 32% (9/28), inflammatory hepatocellular adenoma in 35% (10/28), atypical hepatocellular carcinoma in 18% (5/28), liver metastasis in 11% (3/28) and cholangiocarcinoma in 4% (1/28) of cases. Management included surgical resection in 54% (15/28) and simple follow-up in 46% (13/28).

### Imaging features

A single lesion was present in 75% (21/28) of patients, 14% (4/28) had oligo-lesions (≤ 5 lesions), and 11% (3/28) had multifocal lesions (Table 2). The median tumor size was 22 mm [IQR, 10–80 mm] at diagnosis. All lesions had well-defined margins and presented with lobulated contours in 61% (17/28) and rounded contours in 39% (11/28). None of the lesions had infiltrative tumor margins.

**Table 2:** CT and MRI imaging findings

Imaging findings	Patients (*n* = 28)	Typical LGVNL (*n* = 18)	Atypical LGVNL (*n* = 10)
Tumor size (mm)*	22 (10–80)	27 (11–80)	16 (10–26)
Number of lesions			
Single lesion	21 (75)	16 (89)	5 (50)
Oligo-lesions (≤ 5 lesions)	4 (14)	2 (11)	2 (20)
Multifocal lesions (> 5 lesions)	3 (11)	0 (0)	3 (30)
Tumor margins			
Lobulated	17 (61)	15 (83)	2 (20)
Rounded	11 (39)	3 (17)	8 (80)
T2 hyper-intensity (*n* = 27)	27 (100)	18 (100)	9 (100)
Moderate	7 (26)	1 (6)	6 (67)
Marked	20 (74)	17 (94)	3 (33)
Flow voids	2 (7)	2 (11)	0 (0)
T1 hypo-intensity (*n* = 27)	27 (100)	18 (100)	9 (100)
DWI hyperintensity (*n* = 27)	27 (100)	18 (100)	9 (100)
ADC tumor value (mm^2^/s)^§^	1.9 × 10^−^^3^ ± 0.4	2.1 × 10^−^^3^ ± 0.3	1.7 × 10^−^^3^ ± 0.3
ADC tumor-ratio^§^	1.7 ± 0.4	1.8 ± 0.4	1.5 ± 0.2
Arterial phase enhancement			
Present	28 (100)	18 (100)	10 (100)
Thick continuous peripheral ‘flower petal shape’	18 (64)	15 (83)	3 (30)
Homogenous	10 (36)	3 (17)	7 (70)
Portal venous phase enhancement	28 (100)	18 (100)	10 (100)
Delayed phase enhancement	28 (100)	18 (100)	10 (100)
Hepatobiliary phase hypo-intensity (*n* = 15, 10, 5)	15 (100)	10 (100)	5 (100)
Imaging follow-up			
Number of patients	19 (68)	14 (78)	5 (50)
Lesion growth (*n* = 19)	8 (42)	5 (26)	3 (16)

Continuous variable expressed as * medians, with IQRs in parentheses or ^§^ mean ± standard deviation. Categorical variables are expressed as numbers of patients, with percentages in parentheses

*DWI* diffusion-weighted imaging, *ADC* apparent diffusion coefficient

**Fig. 3:**
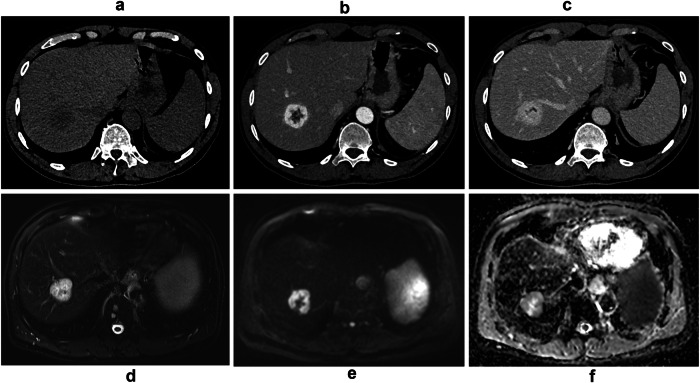
A 58-year-old male with an incidental liver lesion shows the typical LGVNL pattern. **a**–**c** CT scan shows (**a**) hypoattenuating lesion of segment VII, (**b**) thick peripheral lobulated arterial phase “flower petal shape” enhancement, (**c**) progressive centripetal portal phase filling. **d**, **e** On MRI, the lesion shows (**d**) marked T2 hyper-intensity on fat-suppressed T2-weighted image, (**e**) hyper-intensity on high-b value DWI (b = 900) with (**f**) no apparent diffusion coefficient restriction

**Fig. 4:**
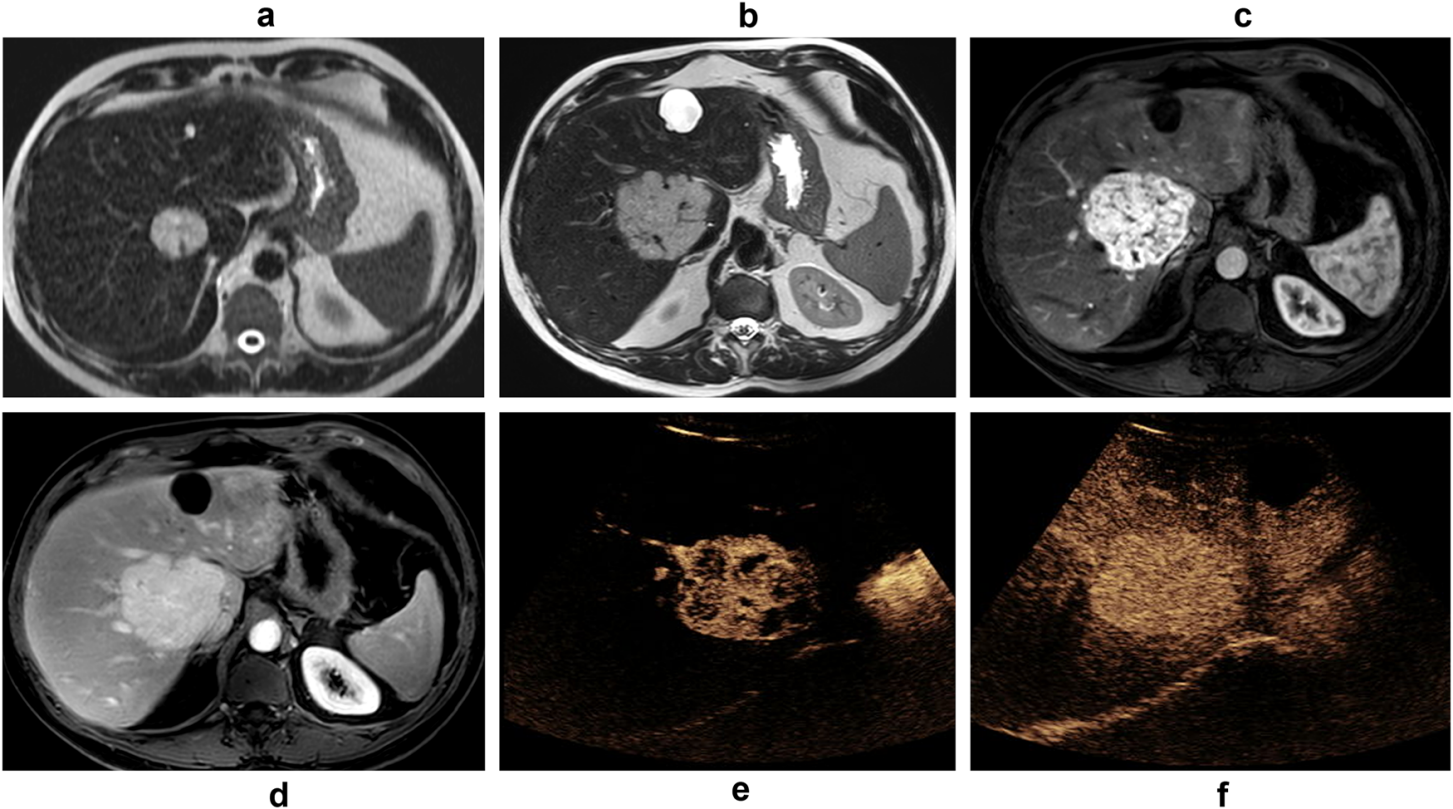
A 47-year-old male showing tumor with typical LGVNL pattern. MRI shows (**a**) hepatic lesion of segment VIII measuring 3.5 cm with marked hyper-intensity on fat-suppressed T2-weighted. MRI follow-up after 8 years (**b**–**d**) demonstrates a significant increase in size. The lesion measures 8 cm with marked T2 hyper-intensity (**b**) and serpiginous signal loss on T2 corresponding to “flow voids,” thick peripheral lobulated “flower petal shape” arterial phase enhancement (**c**) with progressive delayed phase enhancement (**d**). CEUS (**e**, **f**) shows thick peripheral lobulated arterial phase enhancement (**e**) with centripetal portal phase filling (**f**)

**Fig. 5:**
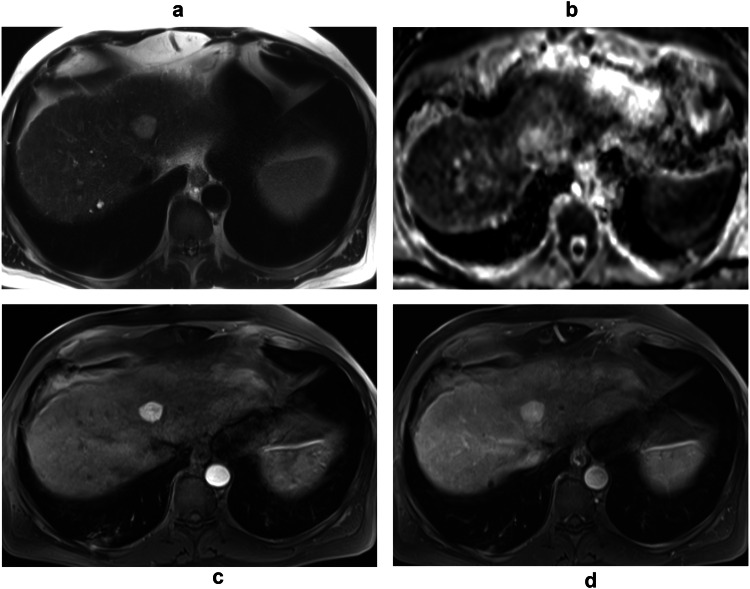
MRI of a 56-year-old male shows an atypical LGVNL pattern. Axial (**a**) T2-weighted image, (**b**) ADC map and (**c**, **d**) contrast-enhanced MRI in the (**c**) hepatic arterial and (**d**) portal venous phases show a lesion of the left lobe with moderate T2 hyper-intensity, no restriction on ADC and homogenous arterial phase enhancement that persists in the portal phase

In the 27 patients who underwent MRI, all lesions were hyperintense on T2-weighted images; 26% (7/27) were mildly hyper-intense and 74% (20/27) were markedly hyper-intense (Figs. 3–5). Flow voids were observed in 7% (2/27) of patients on T2-weighted images and were only noted in large tumors (> 90 mm) (Fig. 4). All lesions were hypo-intense on non-enhanced T1-weighted images. All lesions were hyper-intense on DWI with no ADC restriction. The mean ADC tumor was 1.9 × 10^−3^ ± 0.4 [SD] mm^2^/s and the mean ADC tumor-ratio was 1.7 ± 0.4 [SD]. All lesions showed arterial phase enhancement. Sixty-four percent of these (18/28) showed continuous thick peripheral lobulated enhancement (flower petal shape) with progressive centripetal enhancement and homogenization on portal and delayed phase images (Figs. 3, 4). On the other hand, 36% (10/28) had complete homogeneous arterial phase enhancement that persisted in the portal and delayed phases (Fig. 5). None of our cases showed washout or a capsule during the portal or delayed phases. All lesions were hypo-intense during the hepatobiliary phase. No calcifications, necrosis, hemorrhage or fat-in-lesion were observed. There were no lobar or segmental biliary anomalies or infiltration into vascular structures. All lesions had the same imaging characteristics in patients with multiple lesions.

Ultrasound imaging was performed in 19 cases: 37% (7/19) were hyper-echoic, 21% (4/19) were iso-echoic, and 42% (8/19) were hypo-echoic to the surrounding hepatic parenchyma. Twelve patients underwent CEUS, which showed the same enhancement patterns without washout as the corresponding CT and MRI images (Fig. 4).

Moreover, the results in patients who underwent [^18^F]FDG PET/CT, ^18^F-Choline PET/CT, ^68^Ga-DOTA-TOC PET/CT and ^18^F-FDOPA PET/CT were all negative with an absence of hypermetabolic fixation. The FDG accumulation was the same as in the liver and not like liver cysts without accumulation.

### Typical LGVNL versus atypical LGVNL

Two patterns of LGVNL have been observed on imaging, differentiated by their arterial phase enhancement and significantly associated with certain MRI features (Table 3). The first pattern, called “*typical LGVNL*,” showing a flower petal shape during the arterial phase, was identified in 67% (18/27) of cases on MRI. The second pattern called “*atypical LGVNL*” showing homogeneous arterial phase enhancement was identified in 33% (9/27) of cases on MRI. On univariable analysis, the significant imaging features on MRI of so-called typical lesions versus atypical lesions included: lobulated shape (*p* = 0.001), homogenous and marked hyperintensity on T2-weighted images (*p* = 0.003), with no restriction in the ADC map (ADC tumor value *p* = 0.006 and ADC tumor-ratio *p* = 0.02) (Figs. 3–5). Progressive centripetal enhancement and homogenization on portal and delayed phase images were observed in both patterns of LGVNL.

**Table 3:** Univariable analysis of significant MRI findings of typical LGVNL versus atypical LGVNL patterns

MRI imaging findings	Typical LGVNL (*n* = 18)	Atypical LGVNL (*n* = 9)	Total patients (*n* = 27)	*p*-value
Lobulated shape	15 (83)	1 (11)	16 (59)	0.001
Marked T2 hyper-intensity	17 (94)	3 (33)	20 (74)	0.003
ADC tumor value (mm^2^/s)^§^	2.1 × 10^−^^3^ ± 0.3	1.7 × 10^−^^3^ ± 0.3	1.9 × 10^−^^3^ ± 0.4	0.006
ADC tumor-ratio^§^	1.8 ± 0.4	1.5 ± 0.2	1.7 ± 0.4	0.02

Continuous variable expressed as ^§^ mean ± standard deviation. Categorical variables are expressed as numbers of patients, with percentages in parentheses

*ADC* apparent diffusion coefficient, *LGVNL* low-grade vascular neoplasia of the liver

Logistic regression analysis showed that marked hyperintensity on T2-weighted images was a significant feature independent of age and gender (odds ratio of 41.70, *p*-value = 0.005) (Table 4).

**Table 4:** Logistic regression model for the typical LGVNL pattern

Typical LGVNL aspect		Odds ratio (95% confidence interval)	*p*-value
Age at diagnosis		1.02 (0.93, 1.12)	0.65
Clinical gender male		1.41 (0.11, 17.33)	0.78
Marked T2 hyper-intensity		41.70 (4.34, 1148.73)	0.005

*LGVNL* low-grade vascular neoplasia of the liver

Cohen’s kappa coefficient showed that agreement among the three radiologists for the typical LGVNL pattern was significant for each pair of radiologists, but there was a greater proximity between senior radiologists (Cohen’s kappa = 1.00, *p*-value < 0.001) than between senior and resident radiologists (Cohen’s kappa = 0.515, *p*-value = 0.001).

It was interesting to note that the typical and atypical patterns of LGVNL were not correlated with the histological diagnoses of HSVN and AH: HSVN and AH had typical imaging features in 12 and 6 cases, and atypical features in 8 and 2 cases, respectively (*p*-value = 0.66).

### Long-term progression

Nine of the 28 patients (32%) underwent prompt surgical resection after diagnosis, and 19/28 (68%) were followed up on imaging for a median of 16 months [IQR, 3–120 months]. Six of the latter (32%) patients underwent surgical resection during follow-up. Finally, a total of 15/28 (54%) patients underwent surgical resection with no follow-up imaging analyses after surgery.

**Fig. 6:**
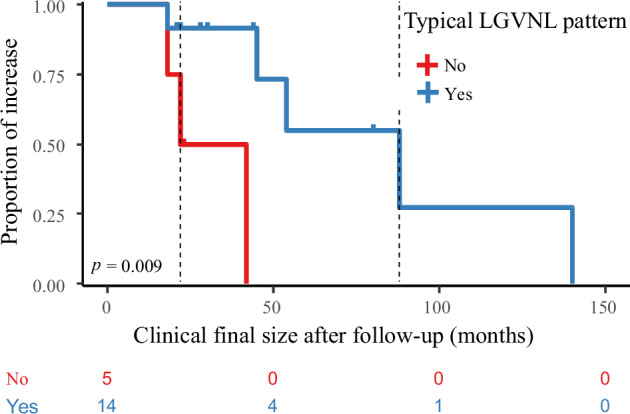
Kaplan–Meier and log rank for time of increase size in months during follow-up and stratified on binary outcome: typical LGVNL pattern on imaging

Follow-up imaging showed that the tumor size increased in 42% (8/19) of patients, with a median increase of 13 mm [IQR, 7–112 mm] (Fig. 4). The largest tumor during follow-up was 140 mm. Time event analysis of an increase in tumor size (mm) during follow-up showed that patients with the “atypical” LGVNL pattern progressed faster than those with the “typical” pattern (median of 32 months versus 88 months, log rank *p*-value = 0.009, Fig. 6). There was no relationship between tumor growth and the histological diagnoses of HSVN or AH. There was no recurrence in patients who underwent complete resection. There were no cases of spontaneous complications such as rupture or hemorrhage or extrahepatic metastases.

## Discussion

The imaging features of LGVNL are not well known, indicated by the limited number of publications and the high rate of misdiagnoses in clinical practice. This series of 28 histologically proven cases of LGVNL, which is the largest reported group in the English-language literature, highlights the main diagnostic imaging features of this entity. Our results show that LGVNL has two distinct imaging patterns. Both patterns share imaging features with other benign vascular lesions: hyper-signal on T2- and diffusion-weighted images with no restriction on the ADC map and persistent delayed phase enhancement. The most common presentation, the typical LGVNL pattern, is a well-defined lobulated lesion with a marked hyper-signal on T2-weighted images and continuous thick peripheral lobulated arterial phase enhancement with progressive centripetal portal and delayed phase enhancement. As described in previous publications, our study confirms the typical imaging feature of LGVNL with a flower petal or sunflower appearance on arterial phase contrast-enhanced images [23]. This typical LGVNL pattern may also include lesion vessel “flow voids” (2\26) as described in vascular malformations, especially if the lesion is large or increasing in size [24]. The second pattern, atypical LGVNL, involves round lesions with a different enhancement pattern, including homogeneous arterial phase enhancement that persists in the portal and delayed phases. There are no calcifications in either pattern, no fat-in-lesion and both patterns are hypo-intense during the hepatobiliary phase. No correlation was found between the typical and atypical imaging features of LGVNL and the histological diagnoses of HSVN and AH.

Knowledge of these imaging features helps to eliminate differential diagnoses. The only differential diagnosis for typical LGVNL is hepatic cavernous hemangioma. However, LGVNL does not present with arterial phase globular discontinuous peripheral enhancement, which is typically found in classic hepatic cavernous hemangioma [25]. The main differential diagnosis of atypical LGVNL is inflammatory hepatic adenoma. In fact, both are hyperintense on T2-weighted images, show complete early arterial enhancement during the arterial and portal venous phases, persistent enhancement over time, as well as a hyposignal during the hepatobiliary phase [26]. It is impossible to distinguish inflammatory hepatic adenomas and atypical LGVNL merely on imaging, unless the ADC of the lesions is considered. Indeed, unlike LGVNL, inflammatory hepatic adenomas show mild diffusion restriction on the ADC map with mean ADC values that are lower than those of the surrounding normal liver parenchyma [27]. In any case, a histological analysis is required to confirm the diagnosis. Also, small hepatic hemangiomas, which may fill rapidly during the arterial phase with immediate homogeneous enhancement, may be difficult to differentiate. However, unlike LGVNL, these lesions are small, less than 2 cm in size, with lesion enhancement similar to aortic enhancement and perfusion disorders during the arterial phase.

Interestingly, as reported in the literature, we observed LGVNL associated with metabolic liver disease or cirrhotic livers in 8/27 (30%) patients [20, 28, 29]. However, because these patients often undergo imaging, incidental discovery of LGVNL in cirrhotic livers may be a bias that may be a diagnostic challenge for the differentiation from malignant tumors, especially atypical hepatocellular carcinoma [29, 30]. However, to our knowledge, washout on portal or delayed phases and the presence of a capsule have never been reported in LGVNL. To differentiate LGVNL from intrahepatic cholangiocarcinoma, the latter usually presents with an absence of arterial phase enhancement as well as with a target sign on DWI and diffusion restriction on the ADC map [31, 32]. In the presence of multifocal hypervascular lesions, it is nearly impossible to differentiate neuro-endocrine liver metastases from multifocal LGVNL lesions on imaging, especially if there is a history of a primary hyperenhancing tumor known to cause hyperenhancing liver metastases. Once again, the map and ADC values can help characterize these lesions [33, 34]. A differential diagnosis with malignant vascular tumors, HEHE and hepatic angiosarcoma is generally possible on imaging [35]. Indeed, HEHE tends to involve the peripheral portions of the liver, resulting in capsular retraction with progressive targetoid enhancement on imaging, while angiosarcoma shows progressive but incomplete enhancement in an irregular flame-shaped pattern [36, 37].

The use of nuclear imaging can help exclude other diagnoses. In our study, certain patients underwent ^18^F-FDG PET/CT, ^18^F-Choline PET/CT, ^68^Ga-DOTA-TOC PET/CT and ^18^F-FDOPA PET/CT and none of the LGVNL tumors showed metabolic uptake with any of the tracers.

Information on the natural progression of LGVNL is limited. Tumor growth was observed in 42% of our cases, with a slower median growth in 88 months in those with typical LGVNL. Based on our observation of natural tumor progression, as well as the reports in the literature, we, like others, suggest that LGVNL be managed by resection after confirmation of the diagnosis by liver biopsy with clinical and radiological follow-up [38].

This study has certain limitations. In particular, the retrospective design with different imaging modalities, and the limited number of patients due to the rarity of this entity. Furthermore, because hepatic cavernous hemangioma, HEHE and hepatic angiosarcomas were not included, there is no comparison between these lesions and the imaging findings of LGVNL. In addition, due to the small sample size of our cohort, we limited the follow-up analyses in the Kaplan–Meier analysis to the univariable level. In conclusion, LGVNL is a group of rare vascular tumors that were recently identified in the adult liver. The typical imaging pattern of LGVNL includes arterial phase “flower petal shape enhancement” with a marked hypersignal on T2-weighted imaging and high ADC, allowing detection and early diagnosis for optimal management of the patients.

## Abbreviations

ADC: Apparent diffusion coefficient
AH: Anastomosing hemangioma
DWI: Diffusion-weighted imaging
HBP: Hepatobiliary phase
HEHE: Hepatic epithelioid hemangioendothelioma
HSVN: Hepatic small vessel neoplasm
LGVNL: Low-grade vascular neoplasia of the liver
SI: Signal intensity
